# Loneliness, social isolation, and health complaints among older people: A population-based study from the “Good Aging in Skåne (GÅS)” project

**DOI:** 10.1016/j.ssmph.2022.101287

**Published:** 2022-11-07

**Authors:** Markus Svensson, Aldana Rosso, Sölve Elmståhl, Henrik Ekström

**Affiliations:** Department of Clinical Sciences in Malmö, Division of Geriatric Medicine, Skåne University Hospital, Lund University, Malmö, Sweden

**Keywords:** Loneliness, Social isolation, Health complaints, Older adults, GÅS, Good Aging in Skåne study, SNAC, Swedish National Study on Aging and Care, HLC, health locus of control

## Abstract

**Purpose:**

To explore associations between perceived loneliness, social isolation, and health complaints among older people.

**Methods:**

5804 participants from the Swedish population study “Good Aging in Skåne” were included. Structured interviews and questionnaires were used to assess perceived loneliness, social isolation, 30 somatic and mental-health related symptoms, socio-demographics, lifestyle, and health. The mentioned symptoms were divided into seven symptom domains: depressive, tension, gastrointestinal- and urinary, musculoskeletal, metabolic, cardiopulmonary, and head-related symptoms. Multiple linear regression was performed to assess associations between reported symptoms and degree of perceived loneliness and social isolation. Multiple logistic regression models were constructed to investigate associations between the prevalence of symptoms in the symptom domains and perceived loneliness and social isolation.

**Results:**

60% of the participants reported feeling lonely at least occasionally. Social isolation was noted by 6%. Higher levels of perceived loneliness were associated to an increased number of reported symptoms. Lonely participants had a higher prevalence of symptoms in all investigated symptom domains, ranging from 67% (gastrointestinal-urinary) to 96% (depressive) for the group experiencing constant loneliness.

**Conclusions:**

Perceived loneliness is a common condition among older people in modern day Sweden and potentially harmful for their subjective well-being and health.

## Highlights

• 60% of the participants reported feeling lonely at least occasionally.
• 6% of the participants were socially isolated.
• Higher levels of perceived loneliness were associated to an increased number of reported symptoms.

## 1 Introduction

Loneliness and social isolation have been associated to a wide variety of negative health consequences, including increased rate of mortality, cardiovascular disease, depression, and dementia (Leigh-Hunt et al., 2017). Although the direction of causality is not fully understood, many pathophysiological mechanisms have been proposed by which loneliness and social isolation may affect health, for example via increased sympathetic neural tone, altered neuroendocrine regulation, reduced sleep quality, and altered immunological response systems (Cacioppo & Hawkley, 2003; Cacioppo et al., 2014; Hawkley et al., 2012).

*Loneliness* can be defined as the subjective experience of lacking desired quantity and/or quality of social relationships (de Jong-Gierveld, 1987; Peplau et al., 1982). In contrast, the term *social isolation* is commonly used to describe the objective (actual) absence of social interactions and relationships (de Jong-Gierveld et al., 2006). Although related, loneliness and social isolation do not always occur simultaneously; lonely people are not necessarily socially isolated and vice versa (de Jong-Gierveld et al., 2006).

It is challenging to estimate the prevalence of social isolation and loneliness. Use of different definitions and cultural differences hamper the external validity of results presented in the literature (Leigh-Hunt et al., 2017). In addition, the prevalence of both loneliness and social isolation changes with age (Dykstra, 2009). Previous studies have estimated the prevalence of loneliness in the age group 60–79 years to be 20–35% and found that 5–12% of the subjects are socially isolated (Dykstra, 2009; Hawthorne, 2008; Iliffe et al., 2007; Ong et al., 2016; Taylor et al., 2018). In the group of people aged 80 years and older, 40–50% are reportedly feeling lonely and 9–21% are regarded as socially isolated (Aoki et al., 2018; Dykstra, 2009; Kobayashi & Steptoe, 2018; Menec et al., 2019; Taube et al., 2013).

Few studies have examined the relationship between perceived loneliness, social isolation, and subjective health complaints (Cacioppo et al., 2006; Gan et al., 2015; Ge et al., 2017; Lee et al., 2021; Taube et al., 2013, 2015), where most examined associations between perceived loneliness and depressive symptoms (Cacioppo et al., 2006; Gan et al., 2015; Ge et al., 2017; Lee et al., 2021). There is limited knowledge whether perceived loneliness and social isolation may affect other forms of health complaints as well. A study of older people in Sweden found an association between perceived loneliness and an increased number of health complaints (Taube et al., 2015). All participants included in the mentioned study were dependent in activities of daily living (ADL) and had a high degree of health care consumption (Taube et al., 2015). Another study reported similar findings, although the study only included people aged 78 years and older (Taube et al., 2013).

A comprehensive assessment of both somatic- and mental health-related symptoms, and separate analyses of specific symptoms or groups of symptoms, are warranted to further elucidate the relationship between perceived loneliness, social isolation, and subjective health among older adults. The aim of this study was therefore to examine associations between perceived loneliness, social isolation, and both somatic- and mental health-related symptoms in the general population of older people. Our sample was obtained from the Swedish cohort study Good Aging in Skåne (GÅS) (Lagergren et al., 2004) and included 5804 individuals aged 60–96 years.

## 2 Methods

### 2.1.1 Study population

**Fig. 1:**
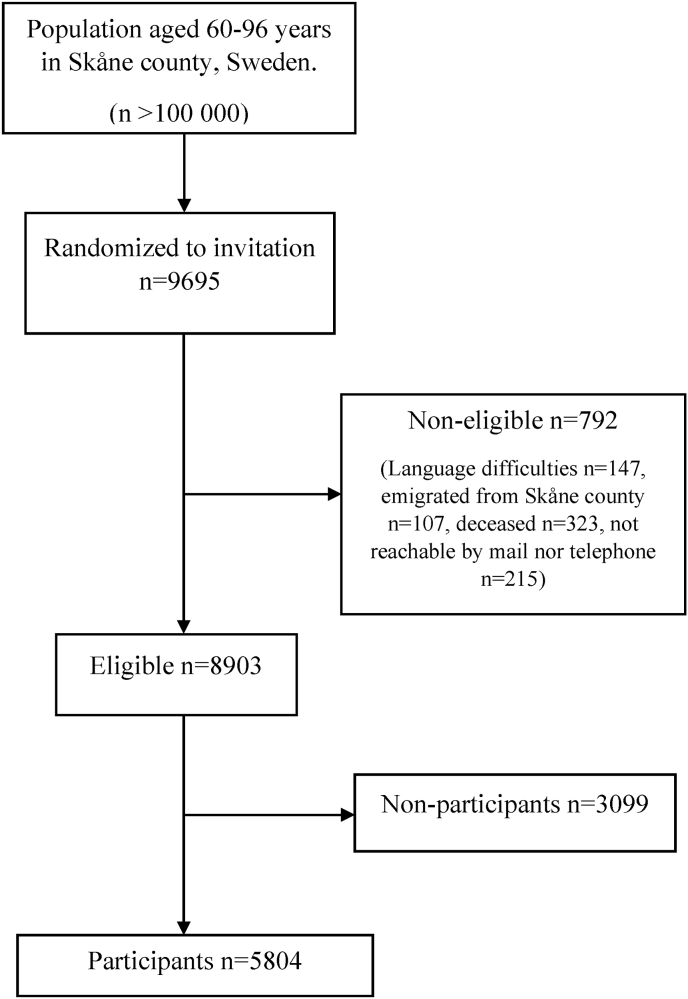
Flow diagram describing the selection of participants for the study.

In this pooled cross-sectional study, participants were drawn from the population study Good Aging in Skåne (GÅS), which is part of the Swedish National Study on Aging and Care (SNAC). The design of the SNAC study is described in more detail elsewhere (Ekström & Elmståhl, 2006; Lagergren et al., 2004). Ten age-cohorts (60, 66, 72, 78, 81, 84, 87, 90, 93, 96-years) were randomly drawn from the population register in five municipalities in the county of Skåne in southern Sweden, covering both urban and rural areas. Three waves were included in this study. Wave 1 recruited participants between 2001 and 2004, wave 2 between 2006 and 2012, and wave 3 between 2012 and 2016. Out of 9695 individuals invited in the three waves, 8903 were eligible and out of those 5804 (65.2%) agreed to participate (Fig. 1).

### 2.1.2 Questionnaires and interviews

Self -reported questionnaires were used to obtain data on socio-demographics, somatic and mental health-related symptoms, perceived loneliness, social isolation, lifestyle, and health. Structured interviews about depression were carried out by medical staff according to predefined research protocols. Assessments took place either at the research centers or, if due to health reasons, in the participants’ homes. The interviews took place between 2001 and 2016.

### 2.1.3 Somatic and mental health-related symptoms

The symptom questionnaire used in this study was a modified version of the Gothenburg Quality of Life instrument (Tibblin et al., 1990). The participants reported if they had experienced any of 30 somatic and mental health-related symptoms during the past three months. The symptom scale has been found to have satisfactory reliability and validity (Sullivan et al., 1993; Tibblin et al., 1990). Symptoms were reported by the participants by answering a Likert-type scale with four possible alternatives: “not at all”, “yes, a little”, “yes, somewhat” and “yes, a lot”. To aid with the interpretation of the results, symptoms were dichotomized into “yes” if participants had experienced the symptom in question to any extent during the past 3 months and “no” if not experienced during the past 3 months. The number of reported symptoms by the participants was constructed into a composite variable, ranging from 0 to 30 symptoms.

The symptoms were also grouped into seven domains (Tibblin et al., 1990). To be categorized into one or more domains of symptoms, a participant should have experienced at least one symptom of the domain in question during the past 3 months (Ekström et al., 2020). Depressive symptoms included tearfulness, depressed mood, general fatigue, sleep disturbance, and exhaustion. Tension symptoms included irritability, nervousness, impaired concentration, difficulty in relaxing, and restlessness. Gastrointestinal- and urinary symptoms included difficulty in passing urine, loss of appetite, nausea, diarrhea, constipation, and abdominal pain. Musculoskeletal symptoms included pain in the joints, backache, and leg pain. Metabolic symptoms included feeling cold, sweating, loss of weight, and being overweight. Cardiopulmonary symptoms included breathlessness, chest pain, and cough. Head symptoms included dizziness, headache, impaired hearing, and eye-problems.

### 2.1.4 Perceived loneliness and social isolation

Perceived loneliness was assessed with a single-item question: “When you look back at the past 3–5 years, which alternative fits you? “I have never once felt lonely”, “I have felt lonely at single occasions”, “I have experienced recurring periods of loneliness”, “I have lived with a constant feeling of loneliness” (Taube et al., 2013). The following information was provided in the questionnaire: “By loneliness we mean the very feeling of being lonely, and not whether you are with other people or not”. Since social isolation is primarily defined, from a Swedish context, as living alone and having infrequent contact with friends and relatives (infrequent contact defined as monthly contact or less frequent) (Government report - Four percent are socially isolated in Sweden, 2019), the social isolation variable used was operationalized as living alone and being in direct contact (physical meeting) with friends or relatives at most monthly (Hawton et al., 2011).

### 2.1.5 Socio-demographics

Socio-demographics included sex, age, education, cohabiting status, financial status, and internal health locus of control (HLC). Level of education was dichotomized into elementary school or below and secondary school or university. Cohabiting status was categorized as cohabiting (married/cohabiting) or living alone. Financial status was assessed by answering “yes” or “no” to the question “has it been difficult to make ends meet for living expenses in the past year?” (Wranker et al., 2018). HLC includes three subscales measuring how individuals believe their health is determined, with one subscale assessing chance HLC (i.e., perceived importance of luck/fate as determinant of health), one subscale assessing internal HLC (i.e., perception of how much the individual themselves control their health), and one subscale for external HLC (i.e., perception of how much other people are responsible for an individual's health) (Wallston et al., 1976). Each subscale includes 6 questions, and the subscales' range is 6–30 points, with high scores indicating high agreement with the statements in the subscale (e.g., high scores on the internal HLC subscale indicate that the individual does to a higher extent believe that they are in control of their own health). In this study we used the internal HLC subscale and the sum scores were dichotomized by the median, dividing participants into high (above the median) or low (below the median) internals (Wallston et al., 1976).

### 2.1.6 Lifestyle habits and depression

Lifestyle variables included smoking habits, alcohol use, and physical activity. Smoking habits were categorized into never smoker, former smoker, or current smoker (Wranker et al., 2019). Alcohol use was categorized into never, 1 to 4 times per month, or ≥2 times per week (Wranker et al., 2018). Physical activity was categorized into mostly sedentary (not more than easier house-hold tasks), lighter activities (activities 2–4 h per week such as walking, gardening, regular house-hold work) and moderate to strenuous activities (exhausting exercise 1–3 h per week like heavier gardening, running, gymnastics or other sports) (Wranker et al., 2019). Depression was assessed by a physician from examination, medical records, and medical history, and categorized as “yes” (depressive episode in the past or present) or “no” (no depressive episode in the past or present or do not know).

### 2.2 Ethics

The study was conducted in accordance with the Helsinki Declaration (World Medical Association Declaration of Helsinki: Ethical Principles for Medical Research Involving Human Subjects, 2013) and approved by the regional ethics committee at Lund University, registration no. LU 744-00. All participants provided a written consent and allowed retrieval of information from the National Board of Health and Welfare national register of In-patient care diagnosis and medical records.

### 2.3 Statistical methods

Prevalence of perceived loneliness and social isolation are presented in Table 1. Differences in perceived loneliness and social isolation by other independent variables were examined with Chi-square (χ2) testing. To explore the distribution of the number of reported symptoms by the independent variables, differences in the number of reported symptoms were investigated with one-way ANOVA or Mann-Whitney *U* test (Appendix Table 1). Perceived loneliness and social isolation may differ by the participants age, sex, and year of birth (i.e., birth cohort effects) (Suanet & van Tilburg, 2019). To examine and quantify these potential differences in the prevalence of perceived loneliness and social isolation, analyses of differences in perceived loneliness and social isolation by age, gender, and wave in the GÅS study were investigated with Chi-square (χ^2^) testing (Appendix Tables 2a and 2b). We hypothesized that perceived loneliness and social isolation would be associated to an increased number of reported symptoms because previous studies have reported a positive association between loneliness and depressive symptoms (Cacioppo et al., 2006; Gan et al., 2015; Ge et al., 2017; Lee et al., 2021). To test this hypothesis, a multiple linear regression model was constructed to explore associations between the overall number of reported symptoms and degree of perceived loneliness and social isolation. Perceived loneliness and social isolation were both included as independent variables in the primary model. Since age, sex, education, alcohol use, smoking, physical activity, and internal health locus of control (HLC) are expected to influence somatic and mental health-related symptoms and perceived loneliness, these factors were also included in the linear model (Table 2). To investigate whether the assumptions of the linear regression model were fulfilled we performed residual analyses. Chi-square (χ2) tests were used to investigate differences in the prevalence of symptoms in each of the seven symptom domains (Table 3). To explore which of the wide range of symptoms that are possibly linked to perceived loneliness and social isolation, multiple logistic regression models were constructed to examine associations of the prevalence of symptoms in the seven symptom domains with perceived loneliness and social isolation as independent variables (Table 4). These models also included age, sex, education, alcohol use, smoking, physical activity, and internal health locus of control (HLC) to minimize confounding.

**Table 1:** Prevalence of perceived loneliness and social isolation. Differences in perceived loneliness and social isolation by other independent variables were examined with Chi-square (χ2) testing.

Variables	Never lonely n (%)	Lonely at single occasions n (%)	Recurring periods of loneliness n (%)	Constant loneliness n (%)	p-value	Not socially isolated n (%)	Socially isolated n (%)	p-value
Whole population	2100 (40.2)	2412 (46.1)	549 (10.5)	169 (3.2)		5297 (94.4)	317 (5.6)	
Age								
60–79 years	1593 (42.2)	1728 (45.8)	364 (9.6)	90 (2.4)	<0.001	3838 (95.9)	164 (4.1)	<0.001
≥80 years	507 (34.8)	684 (47.0)	185 (12.7)	79 (5.4)		1459 (90.5)	153 (9.5)	
Sex								
Male	1203 (50.2)	969 (40.4)	177 (7.4)	48 (2.0)	<0.001	2445 (95.2)	123 (4.8)	0.01
Female	897 (31.7)	1443 (50.9)	372 (13.1)	121 (4.3)		2852 (93.6)	194 (6.4)	
Cohabiting status								
cohabiting	1650 (53.0)	1254 (40.3)	169 (5.4)	40 (1.3)	<0.001	3337 (100)	0 (0) <0.001	
Living alone	449 (21.3)	1155 (54.7)	380 (18.0)	128 (6.1)		1961 (86.2)	315 (13.8)	
Education								
Elementary school or below	943 (41.2)	1017 (44.4)	240 (10.5)	90 (3.9)	0.02	2240 (93.6)	153 (6.4)	0.14
Secondary school or university	1149 (39.3)	1388 (47.5)	305 (10.4)	78 (2.7)		2812 (94.6)	162 (5.4)	
Financial difficulties in the last year								
No	2031 (41.2)	2284 (46.3)	478 (9.7)	142 (2.9)	<0.001	4776 (94.2)	293 (5.8)	0.33
Yes	63 (22.4)	121 (43.1)	71 (25.3)	26 (9.3)		272 (92.8)	21 (7.2)	
Alcohol use								
Never	376 (36.9)	455 (44.6)	128 (12.5)	61 (6.0)	<0.001	978 (90.1)	107 (9.9)	<0.001
1 to 4 times per month	1197 (40.3)	1366 (46.0)	318 (10.7)	86 (2.9)		2890 (95.0)	152 (5.0)	
≥2 times per week	519 (42.4)	581 (47.5)	103 (8.4)	21 (1.7)		1186 (95.4)	57 (4.6)	
Smoking								
Never	848 (39.3)	999 (46.3)	239 (11.1)	73 (3.4)	0.01	2169 (93.9)	142 (6.1)	0.10
Quit smoking	922 (41.9)	1018 (46.3)	205 (9.3)	56 (2.5)		2236 (95.1)	115 (4.9)	
Currently smoking	328 (38.1)	389 (45.2)	105 (12.2)	39 (4.5)		874 (93.6)	60 (6.4)	
Physical activity								
Sedentary	390 (38.4)	415 (40.9)	141 (13.9)	69 (6.8)	<0.001	987 (91.2)	95 (8.8)	<0.001
Lighter	984 (39.9)	1162 (47.1)	254 (10.3)	67 (2.7)		2380 (94.3)	144 (5.7)	
Moderate to strenuous	721 (41.6)	826 (47.7)	153 (8.8)	32 (1.8)		1680 (95.8)	74 (4.2)	
Locus of control								
Low internal	986 (35.0)	1394 (49.5)	342 (12.1)	97 (3.4)	<0.001	2641 (94.0)	170 (6.0)	0.12
High internal	1097 (46.3)	1000 (42.2)	200 (8.4)	70 (3.0)		2243 (95.0)	119 (5.0)	
Depression								
No	1834 (44.9)	1850 (45.3)	303 (7.4)	98 (2.4)	<0.001	3992 (94.8)	219 (5.2)	<0.001
Yes	220 (22.1)	486 (48.9)	221 (22.2)	67 (6.7)		943 (91.7)	85 (8.3)

**Table 2:** Multiple linear regression model with number of symptoms as the dependent variable and loneliness and social isolation as independent variables. The model was adjusted for age, sex, education, alcohol use, smoking, physical activity, and health locus of control.

Variables	Estimate	95% confidence interval	p-value
Loneliness (ref never)			
Single occasions	2.47	2.14 to 2.79	<0.001
Recurring periods	6.03	5.51 to 6.56	<0.001
Constant	6.09	5.23 to 6.96	<0.001
Socially isolated (ref no)			
Yes	−0.12	−0.77 to 0.54	0.73

Abbreviations: ref, reference category.

**Table 3:** Frequencies of participants reporting at least one symptom in the symptom domains of depressiveness, tension, gastrointestinal-urinary tract, musculoskeletal, metabolism, cardiopulmonary or head by degree of loneliness and social isolation. P-values were attained from Pearson chi-square testing. ^a^ p = 0.01–0.05. ^b^ p = 0.001–0.01. ^c^ p < 0.001. ^ns^ non-significant, p > 0.05.

Symptom domain Variables	Depressive n (%)	Tension n (%)	Gastrointestinal-urinary n (%)	Musculoskeletal n (%)	Metabolism n (%)	Cardiopulmonary n (%)	Head n (%)
Loneliness							
Never	1440 (68.8) ^c^	1199 (57.2) ^c^	691 (33.0) ^c^	1406 (67.1) ^c^	1186 (56.6) ^c^	904 (43.2) ^c^	1179 (56.3) ^c^
Single occasions	2053 (85.3)	1821 (75.7)	1127 (46.8)	1804 (75.0)	1642 (68.2)	1232 (51.2)	1618 (67.3)
Recurring periods	527 (96.0)	485 (88.5)	345 (63.1)	460 (83.9)	433 (78.9)	341 (62.2)	456 (83.1)
Constant	162 (96.4)	153 (91.1)	112 (66.7)	146 (87.4)	137 (81.5)	118 (70.2)	132 (79.0)
Socially isolated							
No	4083 (79.2) ^c^	3582 (69.4) ^a^	2202 (42.7) ^c^	3756 (73.4) ^ns^	3301 (65.1) ^ns^	2500 (49.4) ^b^	3234 (64.3) ^c^
Yes	266 (88.7)	223 (74.8)	162 (54.0)	232 (77.3)	204 (68.0)	175 (58.5)	228 (76.0)

**Table 4:** Multiple logistic regression models with the symptom domains as the dependent variables and loneliness and social isolation as independent variables. The models were adjusted for age, sex, education, alcohol use, smoking, physical activity, and health locus of control. ^a^ p = 0.01–0.05. ^b^ p = 0.001–0.01 ^c^ p < 0.001. ^ns^ non-significant, p > 0.05.

Symptom domain Variables	Depressive		Tension		Gastrointestinal-urinary		Musculoskeletal		Metabolism		Cardiopulmonary		Head	
	*OR*	*CI 95%*	*OR*	*CI 95%*	*OR*	*CI 95%*	*OR*	*CI 95%*	*OR*	*CI 95%*	*OR*	*CI 95%*	*OR*	*CI 95%*
Loneliness (ref never)														
Single occasions	2.47^c^	2.12–2.87	2.28^c^	2.00–2.60	1.74^c^	1.54–1.98	1.39^c^	1.21–1.59	1.55^c^	1.37–1.77	1.38^c^	1.22–1.56	1.52^c^	1.33–1.73
Recurring periods	8.99^c^	5.78–13.98	5.36^c^	4.03–7.12	3.13^c^	2.54–3.84	2.25^c^	1.74–2.90	2.43^c^	1.93–3.07	1.99^c^	1.62–2.44	3.41^c^	2.65–4.39
Constant	8.36^c^	3.65–19.14	6.22^c^	3.61–10.73	3.07^c^	2.17–4.34	2.87^c^	1.75–4.72	2.53^c^	1.68–3.83	2.29^c^	1.60–3.27	2.05^c^	1.37–3.09
Socially isolated (ref no)														
Yes	1.15^ns^	0.77–1.70	0.86^ns^	0.64–1.16	1.06^ns^	0.83–1.37	0.86^ns^	0.64–1.16	0.88^ns^	0.67–1.15	1.07^ns^	0.83–1.38	1.05^ns^	0.78–1.41

Abbreviations: ref, reference category. OR, odds ratio. CI, confidence interval.

Sensitivity analyses including the external HLC subscale as a covariate instead of the internal HLC subscale were conducted. In addition, analyses were conducted having the HLC covariate as a continuous variable (instead of dichotomized), having the education covariate as a four-category variable (instead of dichotomized), and having the alcohol consumption covariate as a four-category variable (instead of three categories). Secondly, given the potential intercorrelating relationship between perceived loneliness and social isolation, separate analyses were made that only included social isolation (i.e., without perceived loneliness) as key independent variable in the multivariate models. Thirdly, analyses separating the two components of the social isolation variable (cohabiting status and infrequent contact with friends/relatives) were carried out.

In the statistical analyzes, differences were considered statistically significant if the p-value was <0.05. All analyzes were carried out using SPSS® version 26 (IBM SPSS Statistics for Windows).

## 3 Results

### 3.1 Description of the study sample

In total, 5804 participants were included in this study (Fig. 1). The mean age was 70 years (SD 10.5) and 55% were women. 60% of the participants were lonely at least occasionally and 6% were classified as socially isolated (Table 1). The mean number of reported symptoms was 9 (SD 6.1) (Appendix Table 1). Older participants and women had higher prevalence of perceived loneliness and social isolation compared to younger participants and men (Table 1). The prevalence of perceived loneliness and social isolation was relatively stable across the different waves included in this study (Appendix Table 2b). An exception was the oldest age group (aged 80 years and older), where perceived loneliness and social isolation were slightly less common in the third and second wave compared to the first study wave (Appendix Table 2b).

### 3.2 Perceived loneliness, social isolation, and total symptom burden

In the multiple linear regression model, perceived loneliness was associated with an increased number of reported symptoms. Those with the most frequent feelings of loneliness (i.e., recurring periods or constant loneliness) reported higher number of symptoms compared to those with no perceived loneliness or loneliness only at single occasions (Table 2). This indicates that those with frequent feelings of loneliness have worse subjective health compared to those without perceived loneliness. Social isolation was not statistically significantly associated with the number of symptoms in the primary analysis (Table 2).

### 3.3 Perceived loneliness, social isolation, and somatic and mental health-related symptom domains

The prevalence of symptoms in the different symptom domains (depressiveness, tension, gastrointestinal-urinary tract, musculoskeletal, metabolism, cardiopulmonary, or head) by degree of perceived loneliness and social isolation are presented in Table 3. Those with the most frequent feelings of loneliness (i.e., recurring periods or constant loneliness) had the highest prevalence of symptoms in all symptom domains, ranging from 67% (gastrointestinal-urinary) to 96% (depressive) for the group experiencing constant loneliness. In relative terms, the prevalence in the symptom domains was 1.3 (musculoskeletal) to 2 times (gastrointestinal-urinary) higher among those with recurring periods or constant loneliness compared to those with no perceived loneliness. In the multiple logistic regression models, higher levels of perceived loneliness were consistently associated with increased odds in all seven symptom domains (Table 4). This indicates that those with frequent feelings of loneliness experience a wide variety of symptoms from many different organ systems. Social isolation was not statistically significantly associated with any of the symptom domains in the multiple logistic regression models in the primary analysis (Table 4).

### 3.4 Sensitivity analyses

The results were consistent in the sensitivity analyses including the external HLC subscale as a covariate instead of the internal HLC subscale, and in the analyses having different categorizations of the HLC, education, and alcohol consumption variables (Appendix Tables 3a–3e). In the separate analyses that excluded perceived loneliness, social isolation was associated with an increased number of reported symptoms in the multiple linear regression model (Appendix table 4). This indicates that those classified as socially isolated have worse subjective health, possibly mediated through perceived loneliness. In the multiple logistic regression models without perceived loneliness, social isolation was associated with increased odds in the depressive symptom domain and the gastrointestinal-urinary symptom domain (Appendix table 5). The results suggest that those classified as socially isolated primarily experience more depressive and gastrointestinal-urinary symptoms compared to the non-isolated. For the analyses with the separated components in the social isolation variable (cohabiting status and infrequent contact with friends/relatives), living alone was associated with an increased number of reported symptoms in the multiple linear regression model (Appendix table 6). In the multivariable logistic regression models, living alone was associated with increased odds in the depressive symptom domain and gastrointestinal-urinary symptom domain (Appendix table 7). These results implies that those living alone have worse subjective health compared to those cohabiting.

## 4 Discussion

### 4.1 Importance, interpretation & previous research

The results from this study show that perceived loneliness is prevalent among older people in southern Sweden, with 60% feeling lonely at least occasionally, and that higher levels of perceived loneliness are associated with worse subjective health. Our findings should emphasize that loneliness is common and possibly affects the subjective well-being and health of the general population of older adults in southern Sweden.

The findings from this study are overall in line with previous studies. The association between loneliness and depressive symptoms has been reported in several studies (Cacioppo et al., 2006; Gan et al., 2015; Ge et al., 2017; Lee et al., 2021; Taube et al., 2013, 2015). Underlying mechanisms whereby loneliness affects depressive symptoms may be low self-belief, negative expectations of social interactions, and biological effects of stress response and inflammation (Hawkley & Cacioppo, 2010; Lee et al., 2021). Associations between perceived loneliness and other health complaints in older people, such as cardiopulmonary symptoms, musculoskeletal symptoms and gastrointestinal- and urinary symptoms, are more scarcely investigated. Underlaying mechanisms whereby loneliness affects these symptoms are unclear, but similar processes as for depressive symptoms (e.g., biological effects of stress response and inflammation) possibly play a role (Hawkley & Cacioppo, 2010). In a study on frail older people (frail defined as being dependent in ADL and having a high degree of health care consumption), perceived loneliness was associated with an increased number of health complaints, using a similar assessment of perceived loneliness and health compliant questionnaires as in our study (Taube et al., 2015). What our study adds, is that this association seems to exist not only among frail older adults, but in the general older population (including the healthier, non-frail older adults) as well. The comprehensive assessment of both somatic- and mental health-related symptoms and analyses of specific symptom domains adds further evidence and granularity to the association between perceived loneliness, social isolation, and subjective health. Our findings show that the association of perceived loneliness with subjective health is not limited to depressive symptoms but impacts somatic-related symptoms as well.

Perceived loneliness and social isolation are to some degree related but do not necessarily occur simultaneously. In our study, perceived loneliness was considerably more common than social isolation. Additionally, merely 32 percent of those classified as socially isolated reported recurring periods or constant feelings of loneliness, and 20 percent reported that they never felt lonely (Appendix table 8). These findings should further emphasize the distinctions between the concepts and the importance of considering both perceived loneliness and social isolation when investigating the social well-being of older adults.

The findings that social isolation was associated with health complaints only in models excluding perceived loneliness suggest that perceived loneliness may be a mediator for the association of social isolation with subjective health (Santini et al., 2020). Additional analyses showed that the social isolation component living alone, but not infrequent contact with friends/relatives, was associated with increased number of reported health complaints, and specifically to depressive symptoms and gastrointestinal-urinary symptoms. Gastrointestinal-urinary symptoms and depressive symptoms often occur simultaneously (Wuestenberghs et al., 2022) and social isolation may aggravate depressive symptoms in patients with gastrointestinal disorders (Mikocka-Walus et al., 2022).

The prevalence of both perceived loneliness and social isolation increased with age. The group experiencing the highest degree of perceived loneliness and social isolation were women aged 80 years and older. This finding is in line with previous studies (Dykstra et al., 2005; Kobayashi & Steptoe, 2018; Taube et al., 2013). There are several reasons to why older people experience higher levels of perceived loneliness. When becoming older, the risk of losing a spouse and age-related friends increases. Other aspects associated with old age, such as decline in overall health and loss of function and mobility, also hamper the ability to physically interact with friends and relatives. Compared to men, women are more likely to be widowed (Pinquart & Sörensen, 2001). Women are also more willing to admit being lonely when using direct assessments of perceived loneliness (Pinquart & Sörensen, 2001). This may partially explain why perceived loneliness and social isolation were more prevalent among women compared to men.

Previous studies with prospective designs indicate a longitudinal relationship between loneliness and negative health outcomes, such as cardiovascular diseases (Leigh-Hunt et al., 2017; Steptoe & Kivimäki, 2013). Yet, our study design prevents us from drawing conclusions concerning the direction of association identified between perceived loneliness and subjective health. Reverse causation, were subjective health affects perceived loneliness, is also possible. Longitudinal studies, preferably with an interventional design aiming to reduce loneliness, are warranted to further elucidate the potential role and the direction of association of loneliness in respect to subjective health.

### 4.2 Study strengths

The study sample was randomly drawn from the general population of older people in southern Sweden aged 60–96 years, covering both rural and urban areas. We had a large participation rate (65%), and to further reduce selection bias, home visits were offered for those participants who were unable to visit the study centers. Aid was also offered to participants who had difficulties answering the questionnaires due to language difficulties, visual impairment, or other disabilities. A comprehensive assessment of both somatic- and mental health-related symptoms, and separate analyses of specific groups of symptoms, were used to elucidate the relationship between perceived loneliness, social isolation, and subjective health among older adults.

### 4.3 Study limitations

This study has several limitations. We assessed perceived loneliness by a single-item question. The advantage of single-item questions, compared to multi-item scoring assessments (e.g., loneliness scales), is that they are easily interpreted in clinical and research settings (Luanaigh & Lawlor, 2008). The disadvantages of direct single-item assessments are their unidimensional simplicity and the assumption that the participants understand the concept of loneliness (Luanaigh & Lawlor, 2008). In addition, since both loneliness and social isolation are associated with negative connotations, there is a risk of under-reporting when using self-reported assessments of perceived loneliness and social isolation (Pinquart & Sörensen, 2001). To minimize the mentioned drawbacks, the interviews were done by specially trained personnel, and each task was carefully explained. The loneliness-related questions were answered by the participants while sitting in a quiet room with access to personnel to ask questions if needed and sufficient time to complete the questionnaires was allowed.

The 30 symptoms included in our health complaint questionnaire were classified into seven groups (domains) of symptoms (Tibblin et al., 1990). It is important to note that these symptom domains are not equivalent to any disease nor syndrome. Rather, these symptom domains should be regarded as a novel, theoretical clustering of symptoms. The clinical relevance of such a symptom clustering have not yet been completely determined, and our results should therefore be interpreted with caution from a clinical context.

The strict social isolation classification used made the group “socially isolated” considerably small (6%), although within the prevalence range seen in other studies on similar populations (5–12%) (Hawthorne, 2008; Iliffe et al., 2007; Ong et al., 2016; Taylor et al., 2018). In addition, we did not account for non-physical contacts (e.g., telephone, social media) when assessing social isolation in our study. Non-physical interactions may reduce perceived social isolation among older people (Chen & Schulz, 2016). Therefore, it is possible that non-physical contacts reduce the negative impact of physical isolation on subjective health. Further studies including non-physical contacts are warranted.

Several measures to reduce selection bias were implemented. However, differences between our study population and the general older population remain. The characteristics of those who agreed to participate in the study may differ from those who declined participation (Hernán et al., 2004). Participants in epidemiological studies are generally more likely to have more favorable socioeconomic status, lower prevalence of risk behaviors (smoking, alcohol use) and lower rates of morbidity and mortality compared to nonparticipants (Galea & Tracy, 2007). This may limit the external validity of our results. Furthermore, this study was conducted in southern Sweden, one of the highest-income regions in the world. The results from this study may not be transferable to populations outside of southern Sweden. Replication studies in other regions and countries with different socioeconomical standards are therefore warranted.

## 5 Conclusions

We found that 60% of older people in southern Sweden feel lonely at least occasionally. Perceived loneliness was associated with an increased number of health complaints and was linked to a wide spectrum of symptom domains. Loneliness is a common condition among older people in modern day Sweden and potentially harmful for their subjective well-being and health.

## CRediT authorship contribution statement

**Markus Svensson:** Conceptualization, Data curation, Formal analysis, Writing – original draft, Writing – review and editing. **Aldana Rosso:** Conceptualization, Writing – review and editing, Supervision. **Sölve Elmståhl:** Conceptualization, Funding acquisition, Writing – review and editing, Supervision. **Henrik Ekström:** Conceptualization, Data curation, Formal analysis, Writing – original draft, Writing – review and editing, Supervision.

## Funding

This work was supported by the Swedish 10.13039/501100005348Ministry of Health and Social Affairs, the Skåne Regional Council, and the 10.13039/501100006310Swedish Medical Research Council [grant numbers 2017-01613; 2017-00639].

## Ethical statement

The study was conducted in accordance with the Helsinki Declaration (“World Medical Association Declaration of Helsinki: Ethical Principles for Medical Research Involving Human Subjects,” 2013) and approved by the regional ethics committee at Lund University, registration no. LU 744-00. All participants provided a written consent and allowed retrieval of information from the National Board of Health and Welfare national register of In-patient care diagnosis and medical records.

## Declaration of competing interest

None.

## Data Availability

The authors do not have permission to share data.
