# Supplementary material for: Loneliness, social isolation, and health complaints among older people: A population-based study from the “Good Aging in Skåne (GÅS)” project
Source: SSM Popul Health. 2022 Nov 7;20:101287. doi: 10.1016/j.ssmph.2022.101287 (PMC9649938; doi:10.1016/j.ssmph.2022.101287)
Supplement: Multimedia component 1 [file mmc1.docx]

**Appendix table 1**. Demographics and comparison of symptom burden by age, sex, socioeconomic status, alcohol use, smoking, physical activity, health locus of control, depression, loneliness, and social isolation. P-values were attained from one-way ANOVA^1^ or Mann-Whitney U-test^2^. Abbreviations: SD, standard deviation. q1, first quartile. q3, third quartile.

| Variable | Sample (n=5804) | Number of symptoms  median (q1–q3) mean (SD) | | p-value |
| --- | --- | --- | --- | --- |
| Whole population | 5804 | 8 (4–13) | 9.2 (6.1) |  |
| Age mean (SD) | 69.7 (10.5) |  |  | <0.001^1^ |
| 60-69 years | 3510 (60.5) | 8 (4–13) | 8.5 (6.1) |  |
| 70-79 years | 559 (9.6) | 9 (5–13) | 9.3 (5.7) |  |
| ≥80 years | 1735 (29.9) | 10 (6–15) | 10.6 (5.8) |  |
| Sex |  |  |  | <0.001^2^ |
| Male | 2636 (45.4) | 7 (3–12) | 8.0 (5.8) |  |
| Female | 3168 (54.6) | 10 (5–14) | 10.1 (6.1) |  |
| Cohabiting status |  |  |  | <0.001^2^ |
| Married/cohabiting | 3338 (58.8) | 8 (4–12) | 8.5 (5.9) |  |
| Living alone | 2342 (41.2) | 10 (5–14) | 10.2 (6.2) |  |
| Education |  |  |  | <0.001^2^ |
| Elementary school or below | 2414 (44.8) | 9 (5–14) | 9.9 (6.1) |  |
| Secondary school or university | 2980 (55.2) | 8 (4–13) | 8.7 (5.9) |  |
| Financial difficulties in the last year |  |  |  | <0.001^2^ |
| No | 5085 (94.5) | 8 (4–13) | 9.0 (6.0) |  |
| Yes | 295 (5.5) | 14 (9–19) | 13.6 (6.5) |  |
| Alcohol use |  |  |  | <0.001^1^ |
| Never | 1095 (20.3) | 10 (6–15) | 10.9 (6.3) |  |
| 1 to 4 times per month | 3052 (56.6) | 8 (4–13) | 9.1 (6.0) |  |
| ≥2 times per week | 1244 (23.1) | 7 (4–12) | 8.0 (5.6) |  |
| Smoking |  |  |  | 0.17^1^ |
| Never | 2336 (41.4) | 8 (4–13) | 9.0 (6.1) |  |
| Quit smoking | 2371 (42.0) | 8 (5–13) | 9.1 (5.8) |  |
| Currently smoking | 942 (16.7) | 9 (4–14) | 9.6 (6.4) |  |
| Physical activity |  |  |  | <0.001^1^ |
| Sedentary | 1097 (20.4) | 11 (7–16) | 11.7 (6.3) |  |
| Lighter | 2528 (47.0) | 9 (5–13) | 9.4 (6.0) |  |
| Moderate to strenuous | 1757 (32.6) | 7 (3–11) | 7.5 (5.5) |  |
| Locus of control |  |  |  | <0.001^2^ |
| Low internal | 2820 (54.3) | 9 (5–14) | 9.9 (6.2) |  |
| High internal | 2369 (45.7) | 8 (4–12) | 8.3 (5.8) |  |
| Depression |  |  |  | <0.001^2^ |
| No | 4313 (80.0) | 8 (4–12) | 8.3 (5.7) |  |
| Yes | 1078 (20.0) | 13 (8–17) | 12.9 (6.1) |  |
| Loneliness |  |  |  | <0.001^1^ |
| Never | 2100 (40.2) | 6 (3–10) | 7.0 (5.3) |  |
| Single occasions | 2412 (46.1) | 9 (5–14) | 9.8 (5.8) |  |
| Recurring periods | 549 (10.5) | 13 (9–18) | 13.8 (6.0) |  |
| Constant | 169 (3.2) | 14.5 (10–19) | 14.5 (6.1) |  |
| Socially isolated |  |  |  | <0.001^2^ |
| No | 5297 (94.4) | 8 (4–13) | 9.1 (6.0) |  |
| Yes | 317 (5.6) | 10 (6–14) | 10.8 (6.2) |  |

**Appendix table 2a.** Comparison of prevalence of loneliness and social isolation by age groups and gender. P-values were attained from Pearson chi-square testing.

| Variables | Never lonely n (%) | Lonely at single occasions n (%) | Recurring periods of loneliness n (%) | Constant loneliness n (%) | p-value | Not socially isolated n (%) | Socially isolated n (%) | p-value |
| --- | --- | --- | --- | --- | --- | --- | --- | --- |
|  |  |  |  |  |  |  |  |  |
| Male, 60-79 years | 921 (51.0) | 734 (40.7) | 121 (6.7) | 29 (1.6) | <0.001 | 1850 (96.3) | 71 (3.7) | 0.22 |
| Female, 60-79 years | 672 (34.1) | 994 (50.5) | 243 (12.3) | 61 (3.1) |  | 1988 (95.5) | 93 (4.5) |  |
|  |  |  |  |  |  |  |  |  |
| Male, ≥80 years | 282 (47.6) | 235 (39.7) | 56 (9.5) | 19 (3.2) | <0.001 | 595 (92.0) | 52 (8.0) | 0.10 |
| Female, ≥80 years | 225 (26.1) | 449 (52.0) | 129 (14.9) | 60 (7.0) |  | 864 (89.5) | 101 (10.5) |  |
|  |  |  |  |  |  |  |  |  |

**Appendix table 2b.** Comparison of prevalence of loneliness and social isolation by waves in the GÅS study, stratified by age. Wave_1_ was recruited between 2001-2004, wave_2_ between 2006-2012 and wave_3_ between 2012-2016. P-values were attained from Pearson chi-square testing.

|  | N total | Never lonely n (%) | Lonely at single occasions n (%) | Recurring periods of loneliness n (%) | Constant loneliness n (%) | p-value | Not socially isolated n (%) | Socially isolated n (%) | p-value |
| --- | --- | --- | --- | --- | --- | --- | --- | --- | --- |
| Wave_1_ 2001-2004 60-79 years | 1854 | 743 (40.1) | 873 (47.1) | 191 (10.3) | 47 (2.5) | 0.029 | 1808 (94.9) | 97 (5.1) | 0.01 |
| Wave_2_ 2006-2012 60-79 years | 1074 | 488 (45.4) | 459 (42.7) | 96 (8.9) | 31 (2.9) |  | 1130 (96.6) | 40 (3.4) |  |
| Wave_3_ 2012-2016 60-79 years | 847 | 362 (42.7) | 396 (46.8) | 77 (9.1) | 12 (1.4) |  | 900 (97.1) | 27 (2.9) |  |
|  |  |  |  |  |  |  |  |  |  |
| Wave_1_ 2001-2004 ≥80 years | 845 | 259 (30.7) | 418 (49.5) | 116 (13.7) | 52 (6.2) | <0.001 | 788 (87.6) | 112 (12.4) | <0.001 |
| Wave_2_ 2006-2012 ≥80 years | 265 | 87 (32.8) | 122 (46.0) | 42 (15.8) | 14 (5.3) |  | 289 (94.1) | 18 (5.9) |  |
| Wave_3_ 2012-2016 ≥80 years | 345 | 161 (46.7) | 144 (41.7) | 27 (7.8) | 13 (3.8) |  | 382 (94.3) | 23 (5.7) |  |

**Appendix table 3a.** Sensitivity analysis with the external HLC subscale as a covariate instead of the internal HLC subscale in the multiple linear regression model for the association between number of symptoms and loneliness and social isolation.

Abbreviations: ref, reference category.

| Variables | Estimate | 95 % confidence interval | p-value |
| --- | --- | --- | --- |
| Loneliness (ref never) |  |  |  |
| Single occasions | 2.62 | 2.30 to 2.95 | <0.001 |
| Recurring periods | 6.19 | 5.66 to 6.71 | <0.001 |
| Constant | 6.15 | 5.29 to 7.02 | <0.001 |
| Socially isolated (ref no) |  |  |  |
| Yes | -0.04 | -0.70 to 0.61 | 0.89 |

**Appendix table 3b.** Sensitivity analysis with the internal HLC subscale as a continuous variable as a covariate instead of dichotomized in the multiple linear regression model for the association between number of symptoms and loneliness and social isolation.

Abbreviations: ref, reference category.

| Variables | Estimate | 95 % confidence interval | p-value |
| --- | --- | --- | --- |
| Loneliness (ref never) |  |  |  |
| Single occasions | 2.43 | 2.11 to 2.76 | <0.001 |
| Recurring periods | 6.00 | 5.47 to 6.52 | <0.001 |
| Constant | 6.04 | 5.17 to 6.90 | <0.001 |
| Socially isolated (ref no) |  |  |  |
| Yes | -0.11 | -0.76 to 0.54 | 0.73 |

**Appendix table 3c.** Sensitivity analysis with the external HLC subscale as a continuous variable as a covariate instead of dichotomized in the multiple linear regression model for the association between number of symptoms and loneliness and social isolation.

Abbreviations: ref, reference category.

| Variables | Estimate | 95 % confidence interval | p-value |
| --- | --- | --- | --- |
| Loneliness (ref never) |  |  |  |
| Single occasions | 2.67 | 2.35 to 3.00 | <0.001 |
| Recurring periods | 6.21 | 5.70 to 6.74 | <0.001 |
| Constant | 6.17 | 5.31 to 7.04 | <0.001 |
| Socially isolated (ref no) |  |  |  |
| Yes | 0.04 | -0.61 to 0.69 | 0.91 |

**Appendix table 3d.** Sensitivity analysis with education level categorized in four groups (not completed elementary school, completed elementary school, completed secondary school, or university) as covariate instead of dichotomized (elementary school or secondary school/university) in the multiple linear regression model for the association between number of symptoms and loneliness and social isolation.

Abbreviations: ref, reference category.

| Variables | Estimate | 95 % confidence interval | p-value |
| --- | --- | --- | --- |
| Loneliness (ref never) |  |  |  |
| Single occasions | 2.52 | 2.20 to 2.85 | <0.001 |
| Recurring periods | 6.09 | 5.57 to 6.61 | <0.001 |
| Constant | 6.11 | 5.24 to 6.97 | <0.001 |
| Socially isolated (ref no) |  |  |  |
| Yes | -0.07 | -0.72 to 0.58 | 0.84 |

**Appendix table 3e.** Sensitivity analysis with alcohol consumption categorized in four groups (never, 1-4 times per month, 2-3 times per week, or 4 times per week or more) as covariate instead of three groups (never, 1-4 times per week, or 2 times per week or more) in the multiple linear regression model for the association between number of symptoms and loneliness and social isolation.

Abbreviations: ref, reference category.

| Variables | Estimate | 95 % confidence interval | p-value |
| --- | --- | --- | --- |
| Loneliness (ref never) |  |  |  |
| Single occasions | 2.47 | 2.14 to 2.79 | <0.001 |
| Recurring periods | 6.03 | 5.50 to 6.55 | <0.001 |
| Constant | 6.08 | 5.22 to 6.95 | <0.001 |
| Socially isolated (ref no) |  |  |  |
| Yes | -0.12 | -0.77 to 0.53 | 0.73 |

**Appendix table 4.** Sensitivity analysis where perceived loneliness was excluded from the multiple linear regression model.

Abbreviations: ref, reference category.

| Variables | Estimate | 95 % confidence interval | p-value |
| --- | --- | --- | --- |
| Socially isolated (ref no) |  |  |  |
| Yes | 0.93 | 0.24 to 1.61 | 0.008 |

**Appendix table 5.** Sensitivity analysis where perceived loneliness was excluded from the multiple logistic regression models with the symptom domains as the dependent variables.

^a^ p=0.01-0.05. ^b^ p=0.001-0.01 ^c^ p<0.001. ^ns^ non-significant, p>0.05.

Abbreviations: ref, reference category. OR, odds ratio. CI, confidence interval.

| Symptom domain  Variables | Depressive | | Tension | | Gastrointestinal-urinary | | Musculoskeletal | | Metabolism | | Cardiopulmonary | | Head | |
| --- | --- | --- | --- | --- | --- | --- | --- | --- | --- | --- | --- | --- | --- | --- |
|  | *OR* | *CI 95 %* | *OR* | *CI 95%* | *OR* | *CI 95 %* | *OR* | *CI 95 %* | *OR* | *CI 95 %* | *OR* | *CI 95 %* | *OR* | *CI 95 %* |
| Socially isolated (ref no) |  |  |  |  |  |  |  |  |  |  |  |  |  |  |
| Yes | 1.62^a^ | 1.11-2.38 | 1.16^ns^ | 0.88-1.54 | 1.30^a^ | 1.02-1.67 | 1.01^ns^ | 0.76-1.35 | 1.04^ns^ | 0.80-1.35 | 1.21^ns^ | 0.94-1.56 | 1.26^ns^ | 0.95-1.69 |

**Appendix table 6.** Sensitivity analysis with the components in the social isolation variable (cohabiting status and infrequent contact with friends/relatives) analysed separately in the multiple linear regression model for the number of symptoms.

Abbreviations: ref, reference category.

| Variables | Estimate | 95 % confidence interval | p-value |
| --- | --- | --- | --- |
| Cohabiting status (ref cohabiting) |  |  |  |
| Living alone | 0.68 | 0.33 to 1.02 | <0.001 |
|  |  |  |  |
|  |  |  |  |
| Infrequent contact with friends/relatives (ref no) |  |  |  |
| Yes | -0.061 | -0.50 to 0.38 | 0.79 |

**Appendix table 7.** Sensitivity analysis with the components in the social isolation variable (cohabiting status and infrequent contact with friends/relatives) analysed separately in the multiple logistic regression models with the symptom domains as the dependent variables.

^a^ p=0.01-0.05. ^b^ p=0.001-0.01 ^c^ p<0.001. ^ns^ non-significant, p>0.05.

Abbreviations: ref, reference category. OR, odds ratio. CI, confidence interval.

| Symptom domain  Variables | Depressive | | Tension | | Gastrointestinal-urinary | | Musculoskeletal | | Metabolism | | Cardiopulmonary | | Head | |
| --- | --- | --- | --- | --- | --- | --- | --- | --- | --- | --- | --- | --- | --- | --- |
|  | *OR* | *CI 95 %* | *OR* | *CI 95%* | *OR* | *CI 95 %* | *OR* | *CI 95 %* | *OR* | *CI 95 %* | *OR* | *CI 95 %* | *OR* | *CI 95 %* |
| Cohabiting status (ref cohabiting) |  |  |  |  |  |  |  |  |  |  |  |  |  |  |
| Living alone | 1.41^c^ | 1.20-1.66 | 1.12^ns^ | 0.98-1.28 | 1.16^a^ | 1.02-1.31 | 0.98^ns^ | 0.86-1.13 | 1.11^ns^ | 0.98-1.27 | 1.06^ns^ | 0.94-1.20 | 1.04^ns^ | 0.91-1.20 |
|  |  |  |  |  |  |  |  |  |  |  |  |  |  |  |
| Infrequent contact with friends/relatives (ref no) |  |  |  |  |  |  |  |  |  |  |  |  |  |  |
| Yes | 0.99^ns^ | 0.81-1.21 | 0.99^ns^ | 0.84-1.18 | 1.04^ns^ | 0.88-1.21 | 0.95^ns^ | 0.80-1.14 | 0.90^ns^ | 0.76-1.06 | 0.98^ns^ | 0.84-1.15 | 1.18^ns^ | 0.99-1.40 |

**Appendix table 8.** Comparison of prevalence of perceived loneliness by social isolation.

|  | Never lonely n (%) | Lonely at single occasions n (%) | Recurring periods of loneliness n (%) | Constant loneliness n (%) |
| --- | --- | --- | --- | --- |
| Not socially isolated | 2040 (41.5) | 2260 (45.9) | 477 (9.7) | 143 (2.9) |
| Socially isolated | 59 (20.2) | 140 (47.9) | 70 (24.0) | 23 (7.9) |
